# Azelastine Inhibits Triple-Negative Breast Cancer Cell Viability via an ARF1-Dependent Mechanism

**DOI:** 10.3390/ijms262411849

**Published:** 2025-12-08

**Authors:** Seon Uk Park, Gi Ung Jung, Eun Kyung Paik, Jeong-Yeon Lee, Dong Charn Cho, Hee Kyoung Chung, Hang Joon Jo, Sung Jun Jung

**Affiliations:** 1 Graduate School of Biomedical Science and Engineering, Hanyang Biomedical Research Institute, College of Medicine, Hanyang University, Seoul 04763, Republic of Korea; seonwook.p@gmail.com (S.U.P.); mkgiung@gmail.com (G.U.J.); 2 Department of Biomedical Informatics, Graduate School of Biomedical Science and Engineering, Hanyang University, Seoul 04763, Republic of Korea; ekpaik@kirams.re.kr; 3 Department of Pathology, College of Medicine, Hanyang University, Seoul 04763, Republic of Korea; jy2jy2@hanyang.ac.kr (J.-Y.L.); hc2n@hanyang.ac.kr (H.K.C.); 4 Hanyang Intercollege, Hanyang University, Seoul 04763, Republic of Korea; 5 Department of Biomedical Engineering, Graduate School of Biomedical Science and Engineering, Hanyang University, Seoul 04763, Republic of Korea; 6 Department of Physiology, College of Medicine, Hanyang University, Seoul 04763, Republic of Korea

**Keywords:** triple-negative breast cancer, azelastine, ARF1, HRH1, drug repurposing, golgicide A, cytotoxicity

## Abstract

Triple-negative breast cancer (TNBC) is an aggressive subtype characterized by a lack of targetable receptors, leading to limited treatment options and a critical need for novel therapeutic strategies. This study aimed to evaluate the potential of azelastine, a clinically approved H1-antihistamine, for drug repositioning against TNBC and to elucidate its underlying HRH1-independent mechanism of action. Cell viability assays (CCK-8) were performed on TNBC cell lines (MDA-MB-231 and BT-549) following treatment with azelastine and its major metabolite, desmethyl azelastine. After observing ambiguous clinical associations between HRH1 expression and patient prognosis, HRH1 dependency was assessed through histamine stimulation and HRH1 knockdown (siRNA). Subsequently, the role of ADP-ribosylation factor 1 (ARF1), found to be overexpressed in TNBC and linked to poor prognosis, was investigated using ARF1 knockdown (siRNA), co-treatment with the Golgi-specific brefeldin A-resistance guanine nucleotide exchange factor 1 (GBF1) inhibitor golgicide A (GCA), and co-treatment with the Drp1 inhibitor M-divi 1. Azelastine and desmethyl azelastine potently reduced MDA-MB-231 cell viability in a dose- and time-dependent manner, achieving cell survivals of 61.3 ± 6.1% (30 µM) and 34.9 ± 3.7% (50 µM) for azelastine, and 52.4 ± 12.5% (30 µM) for desmethyl azelastine, respectively, after 72 h, with an IC_50_ of 35.93 µM determined for azelastine in MDA-MB-231 cells. Additionally, azelastine significantly reduced the viability of BT-549 cells. Bioinformatic analysis of clinical datasets revealed HRH1 downregulation in tumors and, functionally, neither histamine stimulation nor HRH1 knockdown mediated azelastine cytotoxicity in cell culture. Importantly, ARF1 expression was significantly upregulated in TNBC and associated with poor prognosis. Co-treatment with GCA, preventing ARF1 activation, restored viability to near-control levels, supporting dependence on the GBF1–ARF1 activation axis of azelastine, whereas the Dynamic-related protein 1 (Drp1) inhibitor M-divi 1 not only partially rescued CCK-8-based cell viability but also normalized azelastine-induced loss of MitoTracker™ Red CMXRos signal and partially preserved (4′,6-diamidino-2-phenylindole) DAPI-based cell density, indicating Drp1-dependent mitochondrial dysfunction. Furthermore, azelastine selectively reduced p-ERK phosphorylation in the cell signaling pathway. Azelastine exerts potent anticancer effects in TNBC cells via an HRH1-independent, ARF1-dependent mechanism that attenuates the Extracellular signal-regulated kinase (ERK)–Drp1 axis, and induces Drp1-dependent mitochondrial dysfunction, independent of its canonical HRH1 receptor function. This ARF1-dependent mechanism provides strong scientific rationale for the drug repositioning of azelastine as an effective therapeutic agent for ARF1-driven TNBC.

## 1. Introduction

TNBC is a highly aggressive subtype lacking estrogen, progesterone, and human epidermal growth factor receptor 2 (HER2), accounting for approximately 15–20% of all breast cancer cases [1,2,3]. It is characterized by high rates of recurrence and distant metastasis, and remains one of the most challenging subtypes to treat due to the lack of effective targeted therapies [4]. Consequently, TNBC patients face a poorer prognosis compared to those with hormone receptor-positive or HER2-amplified tumors. The absence of approved targeted therapies for TNBC underscores the urgent need for novel treatment strategies [5]. Currently, chemotherapy remains the standard treatment option for TNBC, but its effectiveness is limited, especially in advanced stages [6]. As a result, there is increasing interest in identifying new therapeutic agents and molecular pathways, often through strategies like drug repurposing, that could provide alternative treatment options for TNBC patients [1,7].

The histamine system has gained attention in cancer biology. Histamine is a biogenic amine that signals through four well-characterized G-protein-coupled receptors (HRH1–HRH4) [8]. The role of histamine receptor H1 (HRH1) in breast cancer is complex and not fully elucidated. Some studies suggest that elevated HRH1 expression is linked to poor clinical outcome and angiogenesis, while other reports and databases suggest that its significance across different subtypes still needs to be explored [9,10]. Many H1-antihistamines, such as ebastine and terfenadine, have known off-target effects that are independent of their primary histamine-blocking function [11,12]. These findings suggest that the anti-tumor activities of H1-antihistamines may involve non-histamine-mediated mechanisms [12]. Although these findings nominate HRH1 as a potential target in TNBC, its precise role remains a point of interest for further investigation.

In this context, azelastine, a second-generation HRH1 antagonist widely used as an anti-allergy drug, has also been reported to exert anti-proliferative actions in various cancer cells. H1-antihistamines like azelastine are classified as cationic amphiphilic drugs (CADs) due to their specific chemical structure, which includes ionizable hydrophobic aromatic rings and hydrophilic side chains containing amine functional groups. This structure enables them to easily penetrate cell membranes. As weakly basic cationic amphiphiles, CADs readily cross membranes and undergo lysosomal ion-trapping, leading to high intracellular accumulation—a property thought to underlie several anticancer actions [13,14,15]. Beyond its anti-allergic effects, recent studies have shown that azelastine’s anticancer effects are often mediated through its action as a CAD. Notably, previous reports have shown that azelastine strongly inhibited colorectal cancer cell proliferation and mitochondrial fission both in vitro and in vivo by directly targeting ADP-ribosylation factor 1 (ARF1), thereby interfering with downstream oncogenic signaling (IQGAP1–ERK–Drp1 pathway) and it induces anticancer effects through ARF1 targeting in colorectal cancer cells [16,17]. Consistent with this model, ERK-mediated phosphorylation of Drp1 at Ser616 is a well-established trigger of mitochondrial fission, suggesting that an ARF1–ERK–Drp1 signaling axis could operate in TNBC cells [17,18]. These findings reveal that azelastine can target ARF1, a small GTPase involved in vesicular trafficking, rather than HRH1 blockade to suppress tumorigenesis.

ARF1 plays a central role in intracellular trafficking, Golgi structure maintenance, and mitogenic signaling [19,20,21,22], and it has recently emerged as a key regulator of tumor progression, with its therapeutic potential being actively explored [22,23]. The CAD-associated pathway regulating ARF1 activation has been implicated in cancer cell growth, migration, and survival—for example, the CAD antihistamine azelastine directly targets ARF1 and suppresses oncogenic signaling [16]—while ARF1 itself drives (Phosphoinositide 3-kinase) PI3K- and (Protein Kinase B) AKT-dependent migration/proliferation [19]. High ARF1 expression is observed in aggressive breast cancer subtypes, and ARF1 promotes metastatic behavior. For example, Schlienger et al. showed that ARF1 is highly expressed in advanced breast tumors, and that ARF1 overexpression drives epithelial-to-mesenchymal transition, invasion, proliferation, and chemoresistance [23]. Its role in regulating cancer cell migration and invasion is therefore a major research focus [19,22]. Conversely, ARF1 knockdown in breast cancer models impairs primary tumor growth and markedly reduces lung metastases, highlighting its role in cellular trafficking and cancer progression [24]. In fact, ARF1 is the most frequently amplified ARF family member in breast cancer, and its depletion suppresses metastatic dissemination in mice and zebrafish models [24]. The modulation of ARF1 and its downstream signaling, such as the PI3K-AKT pathway, establishes it as a pro-metastatic signaling hub in breast and other cancers [19,22]. Moreover, Drp1 activity and mitochondrial fission have been repeatedly implicated in breast cancer biology, including TNBC, providing a mechanistic bridge between ARF1 signaling and mitochondrial dynamics [25,26].

Here, we examined whether azelastine exhibits anticancer activity in the TNBC cell lines MDA-MB-231 and BT-549, and sought to distinguish whether such effects may arise from HRH1 antagonism or from properties consistent with its classification as a CAD. Our findings suggest that an HRH1 antagonist may display CAD-associated anticancer actions in TNBC, indicating a potential mechanistic basis for considering azelastine within an expanded therapeutic framework. To further delineate this possibility, we used GCA to suppress ARF1 activation and subsequently evaluated whether azelastine engages an ERK–Drp1-linked mitochondrial dysfunction axis downstream of ARF1 [27]. Clarifying these mechanisms may help define whether azelastine, an established clinical agent, could have relevance as a repurposable therapeutic candidate in TNBC, a subtype with limited targeted treatment options.

## 2. Results

### 2.1. Azelastine and Its Metabolite Decrease TNBC Cell Viability

**Figure 1:**
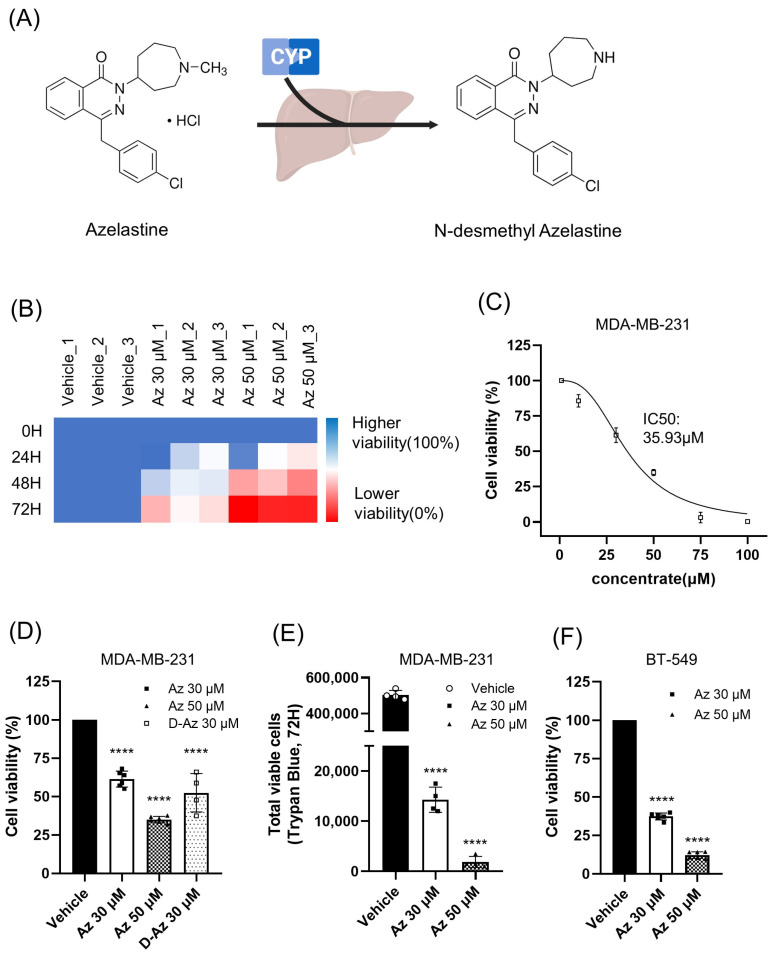
Azelastine and its metabolite potently reduce TNBC cell viability through an HRH1-independent mechanism. (**A**) Azelastine is metabolized in the liver by the CYP450 enzyme to N-desmethyl azelastine. (**B**) Heatmap showing time-dependent reduction in MDA-MB-231 cell viability after treatment with azelastine (30 µM and 50 µM) for 24, 48, and 72 h. (**C**) Dose–response curve of azelastine in MDA-MB-231 cells after 72 h of treatment, showing an IC_50_ of 35.93 µM (**D**) After 72 h, azelastine at 30 µM and 50 µM reduced viability to 61.3 ± 6.1% and 34.9 ± 3.7%, respectively; treatment with 30 µM desmethyl azelastine showed 52.4 ± 12.5% viability. Individual data points are shown using specific markers (■: Az 30 µM, ▲: Az 50 µM, □: D-Az 30 µM). (**E**) Trypan blue exclusion assay showing the number of viable MDA-MB-231 cells after 72 h of treatment. Vehicle-treated cells expanded from the initial seeding density (5 × 10^4^ cells/well) to approximately 5 × 10^5^ viable cells, whereas azelastine at 30 µM and 50 µM almost completely suppressed cell expansion. Individual data points are shown using specific markers (○: Vehicle ■: Az 30 µM, ▲: Az 50 µM). (**F**) Effect of azelastine on BT-549 cell viability after 72 h of treatment. Azelastine at 30 µM and 50 µM reduced viability to 37.2 ± 1.5% and 12.0 ± 0.6% of control, respectively. Individual data points are shown using specific markers (■: Az 30 µM, ▲: Az 50 µM). Data are presented as mean ± SD (*n* = 4–6). Statistical analysis was performed using one-way ANOVA followed by Dunnett’s post hoc test for (**D**–**F**) (**** *p* < 0.0001 vs. vehicle).

To investigate the anticancer effects of azelastine, we performed cell viability assays on TNBC cell lines. Azelastine is primarily metabolized by the hepatic CYP450 enzyme system to its major metabolite, desmethyl azelastine (Figure 1A). We found that both azelastine and its metabolite potently reduced TNBC cell viability.

In MDA-MB-231 cells, treatment with azelastine led to a marked reduction in cell viability in a dose- and time-dependent manner, with the most significant effect observed after 72 h of treatment (Figure 1B). At this time point, 30 µM and 50 µM azelastine reduced cell viability to 61.3 ± 6.1% and 34.9 ± 3.7% of control, respectively (*n* = 6, *p* < 0.0001 for both). Consistent with the dose-dependent reduction, the IC_50_ of azelastine in MDA-MB-231 cells was determined to be 35.93 µM (Figure 1C). Additionally, the metabolite desmethyl azelastine (D-Az) at 30 µM also exhibited significant cytotoxicity, reducing viability to 52.4 ± 12.5% (*n* = 4, *p* < 0.0001) compared to vehicle control (Figure 1D). To verify that the decrease in CCK-8 signal reflected an actual loss of viable cells rather than a change in metabolic activity, we independently quantified viable cell numbers using a Trypan blue exclusion assay after 72 h of treatment. Starting from the same seeding density (5 × 10^4^ cells/well), vehicle-treated cultures expanded to approximately 5.0 × 10^5^ viable cells, whereas 30 and 50 µM azelastine almost completely blocked cell expansion in a dose dependent manner, leaving only ~1.4 × 10^4^ and ~1.5 × 10^3^ viable cells per well, respectively (*n* = 4, *p* < 0.0001 vs. vehicle; Figure 1E).

To further investigate the generalizability of azelastine’s cytotoxic effect, we tested its activity in another TNBC cell line, BT-549. Consistent with the effects observed in MDA-MB-231 cells, azelastine at 30 µM and 50 µM significantly reduced the viability of BT-549 cells to 37.2 ± 1.5% and 12.0 ± 0.6% of control, respectively (*n* = 4, *p* < 0.0001 for both) (Figure 1F).

These results indicate that azelastine exerts significant cytotoxic activity across two genetically and phenotypically distinct TNBC cell lines, suggesting that its anticancer effects may be broadly relevant within heterogeneous TNBC contexts.

### 2.2. HRH1 Is Downregulated in Breast Tumors and Shows an Ambiguous Correlation with Patient Survival

Given that azelastine, a known HRH1 antagonist, exhibits potent cytotoxicity against TNBC cells, the most direct hypothesis is that its anticancer effects are mediated through HRH1 blockade. To evaluate the clinical basis for this hypothesis, we first analyzed HRH1 expression and its prognostic value in breast cancer patient datasets.

**Figure 2:**
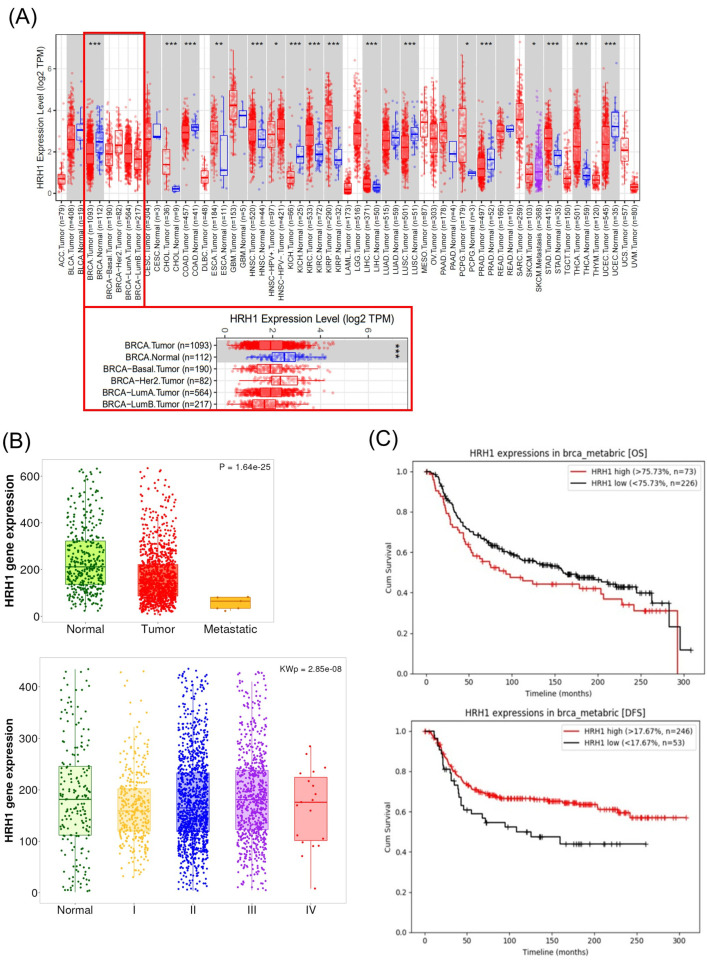
HRH1 expression is downregulated in breast cancer and has ambiguous prognostic value in TNBC. (**A**) Pan-cancer analysis of HRH1 gene expression from TCGA data. Red dots represent Tumor tissues and blue dots represent Normal tissues. HRH1 expression was significantly lower in breast cancer (BRCA) tissues than in normal tissues (* *p* < 0.05, ** *p* < 0.01, *** *p* < 0.001). (**B**) HRH1 gene expression correlates with clinical progression. Expression is significantly lower in tumor and metastatic tissues compared to normal tissue (top; *p* = 1.64 × 10^−25^) and is inversely associated with advanced tumor stages (bottom; Kruskal–Wallis *p* = 2.85 ×10^−8^). (**C**) Kaplan–Meier analysis of TNBC patients (METABRIC dataset) showed that low HRH1 expression was associated with worse disease-free survival (DFS; log-rank *p* < 0.05), but no significant association was observed for overall survival (OS; *p* = 0.1346).

Our analysis of TCGA data revealed a noteworthy finding. A pan-cancer analysis indicated that HRH1 expression is altered in numerous malignancies (Figure 2A). Specifically focusing on breast cancer, RNA-seq data showed that HRH1 mRNA levels were significantly downregulated in tumor tissues compared to normal tissues (*p* < 0.001, Figure 2A). We further examined the relationship between HRH1 expression and clinical progression. As shown in Figure 2B, HRH1 expression was significantly downregulated in primary tumors—and even more so in metastatic samples—compared to normal breast tissue (*p* = 1.64 × 10^−25^). Furthermore, compared to normal tissue, HRH1 expression was significantly lower across all tumor stages (Stage I-IV) (Kruskal–Wallis *p* = 2.85 × 10^−8^).

Moreover, the prognostic value of HRH1 in TNBC patients was ambiguous. According to an analysis of the METABRIC dataset, while lower HRH1 expression was associated with poorer Disease-Free Survival (DFS; log-rank *p* < 0.05), no significant correlation was found with Overall Survival (OS) (log-rank *p* = 0.1346) (Figure 2C).

These clinical findings—the paradoxical downregulation of HRH1 in tumors and its lack of association with overall patient survival—cast doubt on the conventional HRH1 antagonism model. This prompted us to experimentally test whether azelastine’s potent cytotoxicity is indeed dependent on the HRH1 pathway in TNBC cells, as detailed in the following section.

### 2.3. HRH1 Is Functionally Expressed in TNBC Cells but Does Not Mediate Azelastine Cytotoxicity

**Figure 3:**
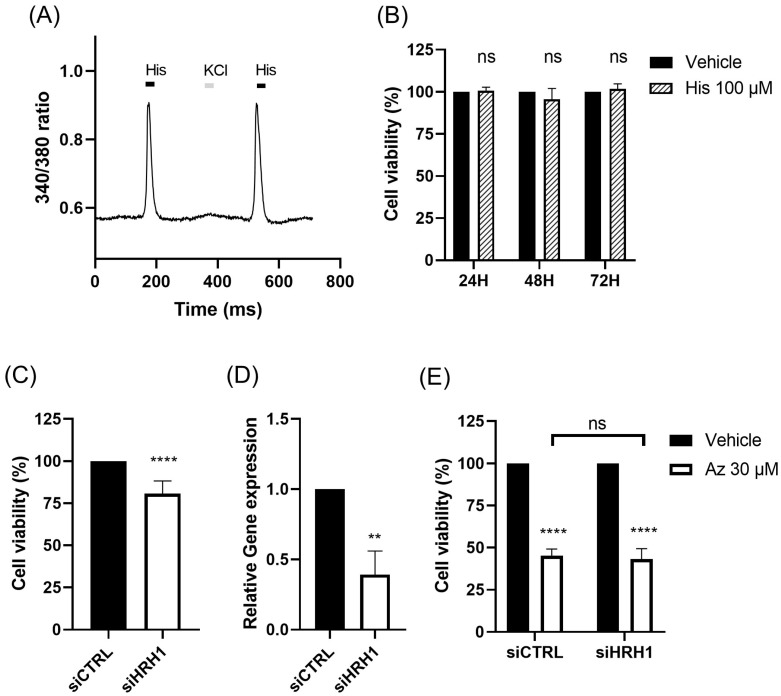
Azelastine-induced cytotoxicity is independent of HRH1 signaling in MDA-MB-231 cells. (**A**) HRH1 functionality was confirmed in MDA-MB-231 cells via calcium imaging after histamine stimulation. Representative calcium flux trace showing transient intracellular calcium rise following histamine (100 µM) stimulation (*n* = 100). (**B**) Cell viability was assessed every 24 h after treatment with 100 µM histamine in MDA-MB-231 cells. Histamine treatment for 72 h does not affect cell viability. (**C**) The effect of HRH1 knockdown on cell viability in MDA-MB-231 cells. (**D**) Relative Gene expression after HRH1 knockdown in MDA-MB-231 cells. (**E**) At 30 µM, azelastine showed a cell viability of 47.37 ± 3.5% in the siCTRL group and 52.26 ± 4.91% in the siHRH1 group, respectively. (ns, not significant; ** *p* < 0.01; **** *p* < 0.0001). Data are presented as mean ± SD. Statistical analyses were performed using unpaired two-tailed Student’s *t*-test for (**C**,**D**) and two-way ANOVA with Tukey’s multiple comparisons test for (**B**,**E**).

The effects of histamine on the MDA-MB-231 cell line were examined to investigate the observed lack of significant correlation between HRH1 expression and survival outcomes in TNBC patients. First, we confirmed the functional expression of HRH1 in MDA-MB-231 cells by assessing their response to histamine (100 µM) stimulation. Calcium imaging experiments were conducted to monitor calcium flux following histamine treatment, and a transient calcium response by histamine was observed in all treated cells, indicating active HRH1 signaling (representative trace; *p* < 0.01 versus vehicle) (Figure 3A). Next, we evaluated the impact of a high concentration of histamine (100 µM) on MDA-MB-231 cell viability. However, treatment of the cells with histamine (100 µM) for 72 h had no significant effect on viability compared to vehicle-treated group. Cell viability was 100.6 ± 2.1% at 24 h, 95.5 ± 6.4% at 48 h, and 101.7 ± 3.1% at 72 h (*n* = 4 per time; two-way ANOVA, ns) (Figure 3B).

Finally, to further elucidate the mechanism of action, we used RNA interference (RNAi) to knock down HRH1 expression in MDA-MB-231 cells. First, we observed the effect of HRH1 knockdown on basal cell viability. HRH1 silencing alone significantly reduced cell viability to 82.1 ± 2.5% compared to the siCTRL group (*n* = 6; unpaired *t*-test, *p* < 0.0001) (Figure 3C), suggesting a potential role for HRH1 in maintaining cell viability. To verify the efficiency of HRH1 silencing, we quantified HRH1 mRNA expression after siRNA transfection. The relative gene expression level was significantly reduced to 0.39 ± 0.16 of control (*n* = 3; unpaired t-test, *p* < 0.01)**,** confirming successful knockdown (Figure 3D). Next, to confirm that its signaling does not mediate azelastine’s action, we assessed azelastine’s effect in these knockdown cells. Despite a reduction in HRH1 expression, azelastine treatment resulted in similar levels of cell viability between the scramble control group (siCTRL, 47.37 ± 3.5% of control, *n* = 6; two-way ANOVA, *p* < 0.0001) and the HRH1 knockdown group (siHRH1, 52.26 ± 4.91% of control, *n* = 6, *p* < 0.0001) (Figure 3E). These findings collectively indicate that while MDA-MB-231 cells express functional HRH1 and its knockdown reduces basal cell viability, this pathway does not mediate azelastine sensitivity. This led us to further investigate the expression and clinical relevance of ARF1 in breast cancer.

### 2.4. ARF1 Is Overexpressed in TNBC and Associated with Poor Patient Survival

**Figure 4:**
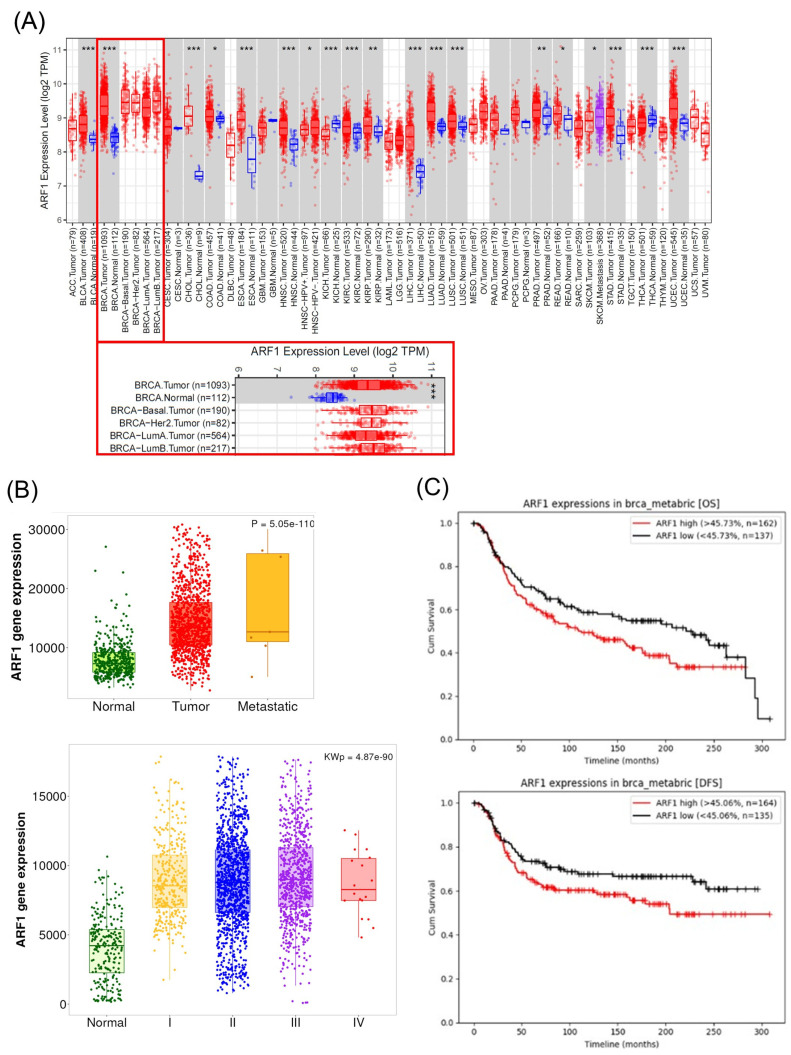
High ARF1 expression correlates with breast cancer progression and poor prognosis in TNBC. (**A**) Pan-cancer analysis of ARF1 gene expression from TCGA data. Red dots represent Tumor tissues and blue dots represent Normal tissues. ARF1 expression was significantly higher in breast cancer (BRCA) tissues than in normal tissues (* *p* < 0.05, ** *p* < 0.01, *** *p* < 0.001). (**B**) ARF1 gene expression correlates with clinical progression. Expression is significantly higher in tumor and metastatic tissues compared to normal tissue (top; *p* = 5.05 × 10^−110^) and shows a positive correlation with advanced tumor stages (bottom; Kruskal–Wallis *p* = 4.87 × 10^−90^). (**C**) Kaplan–Meier analysis of TNBC patients (METABRIC dataset) showed that high ARF1 expression was significantly associated with shorter overall survival (OS) and disease-free survival (DFS) (log-rank *p* < 0.01 for both).

Next, we investigated ARF1 expression across various cancer types using TCGA data. The pan-cancer analysis indicated that ARF1 expression is altered in numerous malignancies (Figure 4A). Specifically focusing on breast cancer (BRCA), RNA-seq data revealed that ARF1 mRNA levels were significantly upregulated in tumor tissues compared to normal breast tissue (*p* < 0.001, Figure 4A).

We further examined the relationship between ARF1 expression and clinical progression. As shown in Figure 4B, ARF1 expression was significantly increased in primary tumors and even more so in metastatic samples compared to normal breast tissue (*p* = 5.05 × 10^−110^). Furthermore, the high expression of ARF1 was not limited to a specific tumor stage. In fact, it remained generally high across all tumor stages compared to normal tissue, with significant differences observed between the stages (Kruskal–Wallis *p* = 4.87 × 10^−90^).

To determine whether the upregulation of ARF1 is associated with survival outcomes in TNBC patients, the METABRIC dataset was used to analyze Overall Survival (OS) and Disease-Free Survival (DFS). Kaplan–Meier analysis demonstrated that high ARF1 expression was significantly associated with poorer Overall Survival (OS) and Disease-Free Survival (DFS) (log-rank *p* < 0.01 for both, Figure 4C). These findings suggest that ARF1 expression may serve as a powerful predictive factor for poor prognosis in TNBC patients.

### 2.5. ARF1 Inhibition Rescues Cells from Azelastine-Induced Death

**Figure 5:**
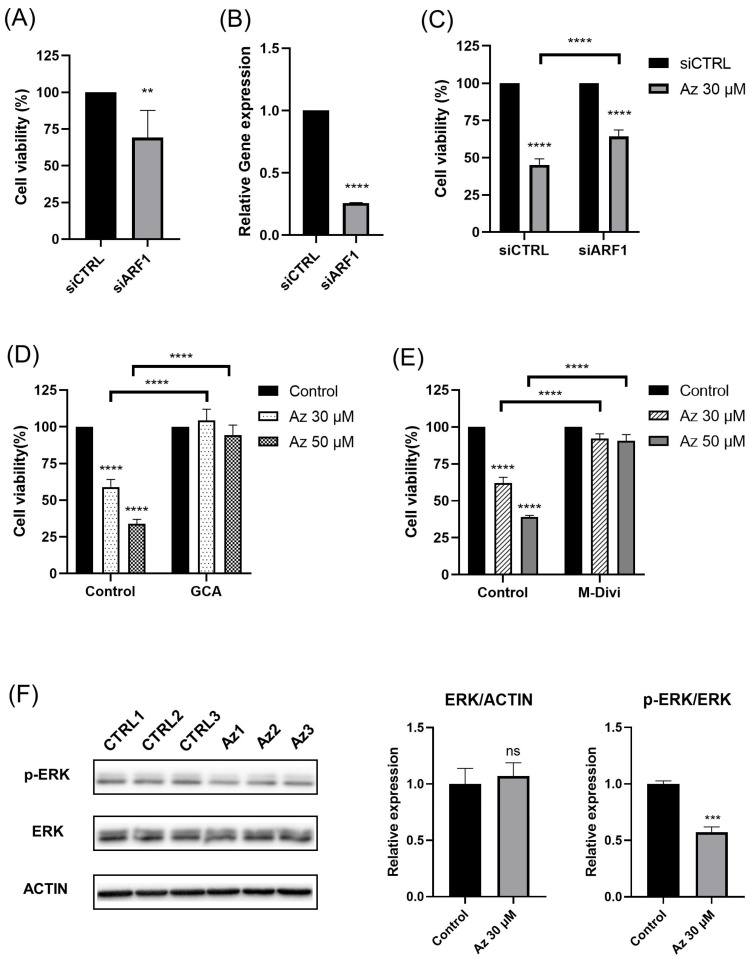
Azelastine induces cytotoxicity in an ARF1-dependent manner and attenuates the ERK-Drp1 signaling axis. (**A**) ARF1 knockdown alone reduced MDA-MB-231 cell viability. (**B**) Relative gene expression after ARF1 knockdown in MDA-MB-231 cells. (**C**) Azelastine treatment (30 µM) reduced cell viability in siCTRL cells to 47.4 ± 3.5%, while in siARF1 cells it was 63.9 ± 4.9%, indicating partial rescue of viability upon ARF1 knockdown. (**D**) Pretreatment with GCA (10 µM), a selective GBF1 inhibitor, significantly attenuated azelastine-induced cytotoxicity, supporting the involvement of ARF1. (**E**) Pretreatment with M-Divi 1 (20 µM), a Drp1 inhibitor, attenuated azelastine-induced cytotoxicity. (**F**) Treatment with azelastine (30 µM, 72 h) reduced ERK phosphorylation in MDA-MB-231 cells. (ns, not significant; ** *p* < 0.01; *** *p* < 0.001; **** *p* < 0.0001). Data are presented as mean ± SD. Statistical analyses were performed using unpaired two-tailed Student’s *t*-test for (**A**,**B**,**F**) and two-way ANOVA with Tukey’s multiple comparisons test for (**C**–**E**).

To investigate whether the anticancer effects of azelastine are mediated through ARF1, we first used RNAi to knock down ARF1 expression in MDA-MB-231 cells. ARF1 silencing alone significantly reduced cell viability to ~75 ± 3.5% (*n* = 6; unpaired *t* test *p* < 0.01) of the control siRNA (siCTRL) group, indicating ARF1’s essential role in TNBC cell proliferation and cell viability (Figure 5A). To confirm the efficiency of ARF1 silencing, we measured ARF1 mRNA expression following siRNA transfection. The relative gene expression level was markedly reduced to 0.25 ± 0.004 of control (*n* = 3; unpaired t-test, *p* < 0.0001)**,** confirming efficient ARF1 knockdown (Figure 5B). We then performed ARF1 knockdown to investigate whether this off-target effect was the primary mechanism of azelastine-induced cytotoxicity. After azelastine treatment, ARF1 knockdown significantly rescued cells from cytotoxicity, with a marked increase in cell viability compared to the siCTRL group (siARF1, 63.9 ± 4.9%; siCTRL, 47.4 ± 3.5%, *n* = 6; two-way ANOVA, *p* < 0.0001, Figure 5C).

Finally, to further elucidate the mechanism of action, we conducted additional experiments using golgicide A (GCA), a GBF1 inhibitor that prevents ARF1 activation. Under the same conditions used to evaluate the viability of MDA-MB-231 cells, the effects of azelastine treated at concentrations of 30 µM and 50 µM for 72 h were compared between cells treated with azelastine alone and those co-treated with GCA. When azelastine was administered alone, cell viability decreased to 58.84 ± 8.5% and 33.83 ± 4.8% at 30 µM and 50 µM, respectively. However, in cells co-treated with GCA, cell viability was restored to 104.27 ± 10.3% and 94.37 ± 8.2% at the same concentrations (*n* = 6; two-way ANOVA, *p* < 0.0001) (Figure 5D). The results demonstrated that GCA restored viability to ~94–104% of control, indicating that ARF1 inhibition restored cell viability to control levels despite azelastine treatment. This significant reversal of azelastine’s anticancer effects upon ARF1 inhibition indicates that ARF1 plays a critical role in mediating the cytotoxic activity of azelastine and these results are consistent with previous findings that azelastine fails to inhibit proliferation in ARF1-deficient cells.

In addition, pretreatment with M-divi 1 (20 µM), a Drp1 inhibitor, significantly rescued MDA-MB-231 cells from azelastine-induced cytotoxicity. After 72 h of treatment, cell viability decreased to 64.97 ± 4.1% with 30 µM azelastine and 40.94 ± 1.1% with 50 µM azelastine compared to vehicle (100 ± 2.7%). In contrast, co-treatment with M-divi 1 increased viability to 92.24 ± 4.8% and 90.70 ± 5.6% at the same concentrations (*n* = 6; two-way ANOVA, *p* < 0.0001). These results indicate that inhibition of Drp1 partially abrogates the cytotoxic effects of azelastine (Figure 5E).

Consistent with these findings, Western blot analysis demonstrated that treatment with azelastine (30 µM, 72 h) selectively reduced ERK phosphorylation without affecting total ERK levels. Quantification showed that the ERK/ACTIN ratio remained unchanged compared with control (1.072 ± 0.117 vs. 1.0 ± 0.139; *n* = 4; ns), whereas the *p*-ERK/ERK ratio was significantly reduced to 0.612 ± 0.048 relative to vehicle controls (1.0 ± 0.026; *n* = 4; unpaired *t* test, *p* < 0.001) (Figure 5F).

These results indicate that the anticancer effects of azelastine are specifically mediated through an ARF1-dependent mechanism.

### 2.6. Drp1 Inhibition Attenuates Azelastine-Induced Mitochondrial Dysfunction and Cell Loss

**Figure 6:**
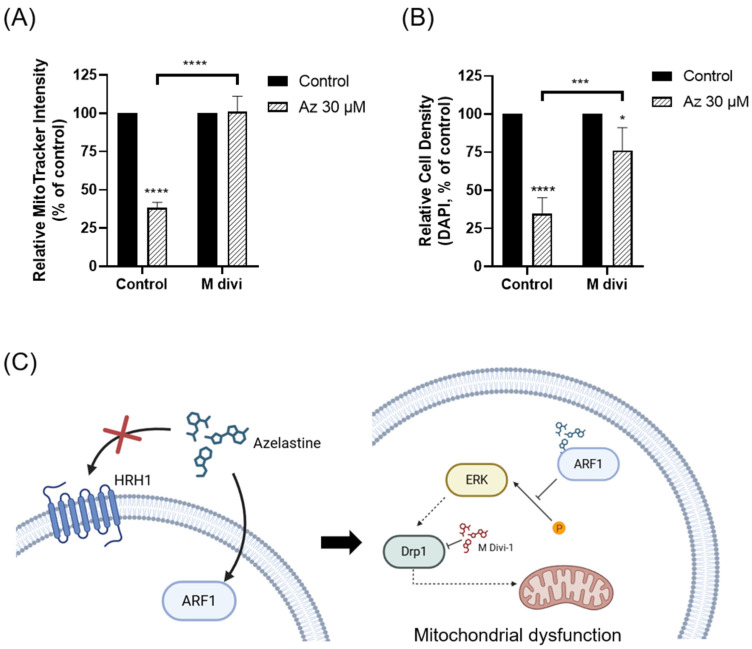
Drp1 inhibition mitigates azelastine-induced mitochondrial dysfunction and cell loss in TNBC cells. (**A**) Relative MitoTracker™ Red CMXRos intensity in MDA-MB-231 cells treated with azelastine (30 µM, 72 h) in the absence (Control) or presence (M-Divi 1) of the Drp1 inhibitor M-Divi 1 (20 µM). Fluorescence was normalized to the corresponding control and expressed as % of control. Azelastine alone markedly reduced mitochondrial signal, whereas co-treatment with M-Divi 1 maintained MitoTracker intensity at control levels. (**B**) Relative cell density assessed by DAPI fluorescence under the same conditions. Azelastine alone decreased DAPI signal, whereas co-treatment with M-Divi 1 significantly increased DAPI fluorescence compared with azelastine alone, indicating partial preservation of cell number. Data are expressed as % of the corresponding control. (**C**) Schematic model illustrating that azelastine enters TNBC cells independently of HRH1 and targets ARF1, leading to suppression of ERK signaling, Drp1-dependent mitochondrial dysfunction, and apoptosis; inhibition of Drp1 by M-Divi 1 attenuates azelastine-induced mitochondrial dysfunction and rescues cell viability. This figure was created using the web-based platform BioRender.com (accessed on 6 November 2025. (* *p* < 0.05; *** *p* < 0.001; **** *p* < 0.0001). Data are presented as mean ± SD. Statistical analysis was performed using two-way ANOVA with Tukey’s multiple comparisons test for (**A**,**B**). In (**C**), solid arrows indicate the direction of signaling/activation, blunt-ended lines denote inhibition (e.g., M-Divi 1–mediated Drp1 inhibition), and dashed arrows represent proposed downstream effects leading to mitochondrial dysfunction and apoptosis.

Next, we examined whether Drp1 inhibition could rescue azelastine-induced mitochondrial dysfunction. After 72 h of treatment, MitoTracker Red intensity in MDA-MB-231 cells was markedly reduced to 38.24 ± 3.60% of vehicle control by 30 µM azelastine (*n* = 4; two-way ANOVA, *p* < 0.0001). In contrast, pretreatment with M-divi 1 (20 µM), a Drp1 inhibitor, almost completely prevented this decrease, maintaining MitoTracker intensity at 101.18 ± 9.90% in azelastine-treated cells (*n* = 4; two-way ANOVA, *p* < 0.0001). These results indicate that Drp1 inhibition effectively rescues azelastine-induced mitochondrial dysfunction (Figure 6A).

To corroborate these findings at the level of cell number, we quantified DAPI fluorescence as a surrogate measure of cell density. Under the same conditions, 30 µM azelastine reduced DAPI signal to 34.73 ± 10.48% of control (*n* = 4; two-way ANOVA, *p* < 0.0001), whereas co-treatment with M-divi 1 increased DAPI fluorescence to 76.14 ± 14.93% (*n* = 4; two-way ANOVA, *p* < 0.0001), indicating a substantial preservation of cell density despite continued azelastine exposure (Figure 6B). Together with the CCK-8 results showing that M-divi 1 restores azelastine-induced reductions in metabolic activity (Figure 5E), these data demonstrate that Drp1 inhibition attenuates azelastine-induced mitochondrial dysfunction and partially abrogates the associated loss of cell viability.

## 3. Discussion

Our findings demonstrate that azelastine, a clinically approved antihistamine, and its metabolite desmethyl azelastine potently inhibit the viability of the TNBC cell lines, MDA-MB-231 and BT-549, through an ARF1-dependent mechanism. Azelastine treatment resulted in a significant, dose- and time-dependent reduction in cell viability, with an IC_50_ of approximately 33 µM in MDA-MB-231 cells. Notably, these cytotoxic effects were unaffected by HRH1 knockdown and were not reproduced by histamine stimulation, indicating that azelastine acts independently of canonical HRH1 signaling. Mechanistically, co-treatment with GCA restored viability to near-control levels, and ARF1 knockdown via RNA interference significantly attenuated azelastine-induced cytotoxicity, increasing cell viability from ~45% in control siRNA-transfected cells to nearly 64% in ARF1-silenced cells. These results indicate that ARF1 activity is essential for azelastine’s cytotoxic effect. Collectively, these findings support the conclusion that azelastine induces cytotoxicity through a non-canonical mechanism involving ARF1 rather than histamine receptor blockade, and may represent a promising therapeutic candidate for TNBC, a disease subtype notorious for its aggressive nature and lack of effective molecularly targeted therapies. Consistent with this mechanism, azelastine markedly reduced mitochondrial signal, and this effect was almost completely prevented by Drp1 inhibition, yielding mitochondrial fluorescence comparable to control levels and indicating that Drp1-dependent mitochondrial dysfunction contributes to the cytotoxic response. Notably, however, the extent of rescue by Drp1 inhibition differed across readouts: while M-divi 1 restored CCK-8-based viability to ~90% of vehicle levels, the corresponding DAPI-based cell density recovered only to ~75% of control, suggesting that Drp1 inhibition normalizes mitochondrial function and metabolic activity in surviving cells more completely than it prevents overall cell loss. This pattern further raises the possibility that Drp1 inhibition slows cell-cycle progression in these cells. In line with this notion, the incomplete recovery of DAPI-based cell density compared with the near-normalization of the CCK-8 signal may reflect reduced proliferative expansion under Drp1 inhibition; Drp1 has been implicated in mitotic fission and efficient cell-cycle progression, and both genetic Drp1 loss and M-divi 1 treatment have been reported to induce cell-cycle arrest and impair proliferation in several cancer models [28]. Together with the observed decrease in p-ERK/ERK and the partial rescue by a Drp1 inhibition, these data support an ARF1-ERK-Drp1 signaling axis underlying azelastine’s anticancer activity in MDA-MB-231 cells and underscore the robustness of this mechanism across multiple functional assays in TNBC cells [16].

The role of HRH1 in breast cancer remains complex, and our findings reaffirm its ambiguous prognostic value. Clinical data showed that HRH1 expression is generally downregulated in tumors and, in TNBC patients, correlated with DFS but not OS, highlighting its uncertain prognostic significance [11,12]. This ambiguity is consistent with our experimental results. Although MDA-MB-231 cells functionally express HRH1, neither HRH1 knockdown nor histamine stimulation altered the cytotoxic effect of azelastine, indicating that its anticancer activity is not mediated through HRH1 signaling. Similar observations have been reported for other H1-antihistamines. For example, ebastine suppresses metastasis in TNBC by targeting focal adhesion ki-nase independently of HRH1 blockade [29]. Such receptor-independent, “off-target” effects may reflect interactions with intracellular signaling proteins, under scoring the broader therapeutic potential of this drug class [11,12]. Our findings further support this concept, demonstrating that azelastine’s efficacy in TNBC relies on ARF1 up stream of the ERK-Drp1-driven mitochondrial dysfunction pathway, rather than on canonical histamine receptor inhibition [16]. Together, these results suggest that the anticancer activity of H1-antihistamines is better explained by their shared properties as CADs, enabling interactions with intracellular targets such as ARF1, rather than by HRH1 blockade. Accordingly, the therapeutic effect of azelastine in TNBC is best understood as an HRH1-independent mechanism driven by the ARF1-ERK-Drp1 axis. Importantly, prior work in other cancer models has shown that azelastine can directly bind ARF1 (DARTS-based target identification and SPR with low-nanomolar Kd; loss of binding in an ARF1 mutant), which is mechanistically congruent with our ARF1-dependency and GCA rescue in TNBC.

While our data strongly support an ARF1–ERK–Drp1 axis as a major downstream mechanism of azelastine-induced cytotoxicity, a key question remains regarding the initial trigger: how does azelastine, an HRH1 antagonist, initiate ARF1 inactivation in an HRH1-independent manner? Azelastine belongs to the class of CADs. CADs are characterized by their lipophilic and basic properties, which cause them to accumulate in acidic organelles, notably the lysosomes, via ion-trapping. This massive accumulation can disrupt lysosomal integrity and function, leading to a state of lysosomal storage disorder-like stress. As a CAD, azelastine is expected to undergo lysosomal ion-trapping and accumulate within acidic organelles, perturbing pH and ionic homeostasis; in line with canonical CAD biology, such accumulation can trigger lysosome-dependent cell death via P2RX4-mediated lysosomal Ca^2+^ release and downstream cAMP signaling [13]. Although not in TNBC, azelastine has been reported in HeLa cells to increase lysosomal cathepsin D/L activities and to induce marked vacuolization/autophagy—changes consistent with lysosomal stress [30]. We therefore posit a CAD → lysosome → ARF1 model in TNBC, wherein azelastine’s cationic amphiphilic behavior precipitates lysosomal stress that converges on ARF1 dysregulation and subsequent attenuation of ERK–Drp1 signaling; this hypothesis will be tested with dedicated lysosomal readouts (LysoTracker/LAMP1, TFEB nuclear translocation, lysosomal pH, and phospholipidosis markers) in future work.

Azelastine is primarily metabolized in the liver by the CYP450 enzyme system to form its major metabolite, desmethyl azelastine. While both compounds function as HRH1 antagonists, desmethyl azelastine is known to have a lower binding affinity for the receptor compared to the parent drug, azelastine. To determine if the observed anticancer effects were primarily driven by azelastine or its metabolite, we assessed the cytotoxic potential of both compounds. Our study demonstrates that despite azelastine’s higher binding affinity for HRH1, both compounds exhibit similar cytotoxic effects on TNBC cells (Figure 1D). This observation, which is not directly correlated with HRH1 binding affinity, supports our hypothesis that the anticancer effects are mediated through a mechanism independent of HRH1 antagonism.

Beyond its role as an HRH1 antagonist, both azelastine and desmethyl azelastine are likely to have similar cell membrane permeability as CADs, defined by a hydrophobic ring system and a hydrophilic cationic amine. When azelastine is metabolized to desmethyl azelastine, only a methyl group is removed from the amine; this demethylation does not alter the key structural features that allow both compounds to function as CADs. These properties allow CADs to accumulate in lysosomes and potentially induce phospholipidosis, a common off-target effect [13,15,31]. Desmethyl azelastine retains both the cationic amine and the hydrophobic aromatic rings, suggesting that it also fulfills CAD structural criteria. Therefore, we hypothesize that the observed anticancer effects of both azelastine and desmethyl azelastine are mediated through their shared CAD properties, rather than exclusively through HRH1 antagonism. Our data suggest that both compounds, owing to these structural similarities, likely contribute to the overall therapeutic response.

Regarding azelastine’s action as a CAD, our data identifies ARF1 as the critical mediator of azelastine’s cytotoxicity. Importantly, RNAi-mediated ARF1 knockdown partially rescued cell viability despite continued azelastine exposure, reinforcing the view that ARF1 is essential for mediating azelastine’s anti-TNBC effects. This observation is consistent with ARF1’s pro-tumorigenic roles in various cancers. While the functions of ARF family members can be context-dependent, ARF1 is frequently implicated in processes that drive malignancy, including cell migration, invasion, and chemo-resistance [19,23,24]. Aligning with this predominant view, our analysis of patient datasets did not show a favorable correlation; on the contrary, we found that high ARF1 expression was significantly associated with worse overall and disease-free survival in TNBC patients [23,24]. This clinical finding provides a strong rationale for our experimental results, where pharmacological inhibition of ARF1 almost completely abrogated azelastine’s cytotoxic effects. Thus, our study supports the view that ARF1 is not only a marker of poor prognosis but also a key functional dependency for TNBC cell survival, making it a compelling therapeutic target. In this context, azelastine’s ability to act upstream of ERK–Drp1 through ARF1 may offer mechanistic breadth compared with Drp1-centric agents, which must balance efficacy against physiological mitochondrial fission.

To contextualize clinical relevance within the evolving TNBC landscape, we briefly compare our mechanism with recent standards. Over the last five years, neoadjuvant/adjuvant pembrolizumab combinations have improved pathologic complete response and event-free survival in early TNBC [32]; PD-1 blockade (e.g., pembrolizumab) has improved outcomes in PD-L1-positive metastatic disease [33]; adjuvant olaparib has conferred overall-survival benefit in gBRCA-mutated, HER2-negative high-risk breast cancer [34]; and the TROP-2-targeting antibody–drug conjugate sacituzumab govitecan has become a standard in pretreated metastatic TNBC [35]. In contrast to these biomarker-defined approaches (PD-L1, gBRCA, TROP-2), azelastine targets a broadly leveraged signaling hub (ARF1 → ERK → Drp1) [16,36]. This positions azelastine as a repurposable, mechanism-based agent that could complement current therapies—either as a biomarker-refined option in ARF1-high tumors or as a rational partner for combinations that impose convergent stress on ERK–mitochondrial fission pathways [37].

Mechanistically, our results in TNBC are consistent with recent reports in other cancer types. A pivotal study demonstrated that azelastine can directly bind ARF1, disrupting its activity and downstream oncogenic signaling, such as the ERK and PI3K/AKT pathways [16,19]. The parallel between our TNBC results and prior studies in other cancers supports the notion that ARF1 is a conserved and druggable vulnerability across cancer types. Future studies should interrogate the relevance of the reported azelastine–ARF1 binding site in TNBC models (e.g., rescue with binding-site mutants) and further characterize downstream signaling (p-ERK, p-Drp1 Ser616).

A limitation of our study is that, even at the high concentration tested (50 µM), azelastine did not completely abolish TNBC cell viability, with a substantial fraction of cells remaining viable (Figure 1D,E). This raises questions about the clinical feasibility of directly translating our in vitro concentrations to patients. Indeed, pharmacokinetic studies indicate that approved oral and intranasal regimens of azelastine yield free plasma concentrations in the low nanomolar range, which are substantially lower than the micromolar doses used here [38]. However, this direct comparison between peak plasma levels and nominal bath concentrations is intrinsically conservative for CADs, because their pharmacology is driven by progressive lysosomal accumulation within acidic compartments rather than by transient free plasma levels. In conventional, relatively short-term in vitro assays, it is technically difficult to fully reproduce this chronic, compartmentalized accumulation that occurs over years of exposure in patients; consequently, higher nominal extracellular concentrations are required to approximate the intralysosomal exposures achieved in vivo. Consistent with this view, the U.S. prescribing information for azelastine hydrochloride nasal spray reports embryo–fetal and maternal toxicities in rodents only at very high oral doses (~68.6 mg/kg/day), corresponding to several-hundred-fold the maximum recommended human daily intranasal dose on a mg/m^2^ basis [39]. These data suggest that azelastine can be administered at exposures that permit substantial tissue and lysosomal accumulation while remaining within an acceptable systemic safety margin in preclinical models. Nonetheless, our findings should be interpreted as proof-of-concept evidence that ARF1 and the ERK–Drp1 signaling axis associated with mitochondrial dysfunction constitute a pharmacologically tractable vulnerability in TNBC, rather than as support for azelastine monotherapy as a stand-alone curative approach. Given that many CAD antihistamines function that enhance the activity of established cytotoxic or targeted agents, future work should evaluate azelastine in rational combination regimens—for example with taxanes, anthracyclines, platinum compounds, or PARP inhibitors—to determine whether ARF1-dependent mitochondrial and lysosomal stress can be leveraged to lower the effective dose, overcome drug resistance, and achieve more profound tumor control than is possible with azelastine alone [11,12,29,30].

Despite these limitations and pharmacokinetic constraints, another important implication of our study is that it delineates the CAD–ARF1 signaling axis as a potential therapeutic target in TNBC. Previous work has implicated CADs in the activation of ARF1 and regulation of tumor cell survival [11,13], and independent studies have established ARF1 as a driver of growth, migration and metastasis in breast cancer [19,23,24]. Our data suggest these lines converge in TNBC. Further studies are warranted to explore how azelastine interacts with CAD-associated effectors and whether this interaction can be optimized. Given azelastine’s clinical familiarity, the pathway lends itself to rapid translational exploration, including biomarker-guided designs that enrich for tumors with high ARF1 expression and prospective testing of combinations (e.g., chemotherapy, ERK-pathway agents, or ADCs).

Given its established safety profile as an FDA-approved antihistamine, azelastine represents a strong candidate for drug repurposing, a strategy with significant logistical and economic advantages [7]. Our findings suggest that TNBC tumors with high ARF1 expression may be especially responsive to this approach [23,24]. This strategy fits within a broader trend of repositioning histamine receptor antagonists for cancer therapy [11,12]. These findings, together with our own, support the idea that antihistamines may harbor unexplored anticancer potential via structural features that enable engagement with oncogenic signaling proteins. From a drug development standpoint, our results underscore the promise of repurposing azelastine as an ARF1-targeting therapy for TNBC. This approach may be rapidly translated into clinical settings. Since azelastine has already passed safety evaluation in humans, early-phase trials in TNBC could be expedited. Moreover, the correlation between ARF1 expression and drug responsiveness suggests that patient stratification based on ARF1 status could enhance therapeutic precision. Prospective validation of ARF1 as a predictive biomarker—together with pharmacodynamic readouts (p-ERK, p-Drp1)—will be central to such designs.

Despite these compelling findings regarding the ARF1–ERK–Drp1 axis, we acknowledge several limitations that should guide future investigations. Firstly, while our study provides robust mechanistic evidence based on in vitro models, a critical next step is the rigorous evaluation of azelastine’s anti-tumor efficacy in vivo using established xenograft models to realize its therapeutic potential in TNBC. Secondly, we recognize the need for enhanced molecular specificity; although our data using the pharmacological inhibitor M-divi 1 strongly support Drp1’s role, its known non-specificity warrants genetic validation (e.g., DRP1 knockdown) and assessment of Drp1 Ser616 phosphorylation to definitively substantiate the proposed axis [40]. Finally, while we propose that azelastine’s Cationic Amphiphilic Drug property acts as the upstream trigger, the direct molecular linkage between this property and ARF1 inactivation requires further experimental confirmation through detailed lysosomal assays. Accordingly, we plan in vivo efficacy and PK/PD studies (orthotopic/xenograft), DRP1 genetic perturbation with measurement of p-Drp1 Ser616 and p-ERK, and a suite of lysosomal assays to bridge CAD behavior to ARF1 inactivation in TNBC.

In conclusion, our study provides compelling evidence that azelastine inhibits TNBC cell viability through an ARF1-dependent mechanism, independent of HRH1 signaling. This effect appears to be mediated via the CAD → ARF1 → ERK → Drp1 axis, underscoring a novel therapeutic pathway in aggressive breast cancers (Figure 6). By repurposing azelastine to target ARF1, we propose a promising strategy to expand treatment options for TNBC patients. These findings also advance our understanding of antihistamine in oncology and highlight ARF1 as a clinically relevant therapeutic target. Further research is needed to validate this approach in vivo and to explore the broader applicability of ARF1-targeted interventions across cancer types. Overall, our expanded data across two TNBC models (MDA-MB-231 and BT-549), together with prior evidence of direct azelastine–ARF1 interaction, nominate ARF1 as a tractable vulnerability in TNBC and support biomarker-guided, mechanism-based development of azelastine in this setting.

## 4. Materials and Methods

### 4.1. Cell Culture

The TNBC cell line MDA-MB-231 was purchased from the Korean Cell Line Bank (KCLB, Seoul, Korea). The cells were cultured under standard conditions at 37 °C in a humidified atmosphere containing 5% CO_2_. According to the supplier’s recommendations, the cells were maintained in Dulbecco’s Modified Eagle’s Medium (DMEM; Gibco, Thermo Fisher Scientific, Waltham, MA, USA) supplemented with 10% fetal bovine serum (FBS; Gibco) and 1% penicillin-streptomycin.

### 4.2. Drug Treatment and Reagents

Azelastine hydrochloride was purchased from Sigma-Aldrich (St. Louis, MO, USA) and dissolved in DMSO to prepare 100 mM stock solutions, which were diluted to final concentrations of 30 µM and 50 µM in culture medium. Histamine dihydrochloride (100 µM; Sigma-Aldrich, St. Louis, MO, USA) and golgicide A (10 µM, GBF1 inhibitor that prevents ARF1 activation; Selleckchem, Houston, TX, USA), M-divi 1 (20 µM, Drp1 inhibitor; Tocris, Minneapolis, MN, USA) were used in designated experiments. Final DMSO concentration was kept below 0.1% in all treatment groups, including vehicle controls.

### 4.3. Cell Viability Assay

#### 4.3.1. Cell Counting Kit-8

Cell viability was measured using the Cell Counting Kit-8 (CCK-8; Dojindo Laboratories, Kumamoto, Japan) following the manufacturer’s protocol. Briefly, MDA-MB-231 cells were seeded at 2 × 10^3^ cells/well in 96-well plates and incubated for 18 h to allow stable attachment. After drug treatment for 24, 48, and 72 h, 10 µL of CCK-8 reagent was added to each well and incubated for 2 h at 37 °C. Absorbance was measured at 450 nm using a microplate reader (iMark Microplate Reader; Bio-Rad, Hercules, CA, USA). Results were expressed as percentages relative to vehicle-treated controls.

#### 4.3.2. Trypan Blue Exclusion Assay

MDA-MB-231 cells were seeded in 6-well plates at 5 × 10^4^ cells/well and allowed to adhere for 18 h before drug treatment. Cells were then treated with azelastine for 72 h. After treatment, cells were detached using 0.25% trypsin–EDTA (Welgene, Gyeongsan-si, Korea), gently resuspended in PBS, and mixed 1:1 with 0.4% trypan blue solution (Sigma-aldrich, St. Louis, MO, USA) to obtain a trypan blue-stained cell suspension. The stained cell suspension was loaded onto a hemocytometer (Marienfeld superior, Lauda-Königshofen, German) and counted under a light microscope (Olympus IMT-2, Tokyo, Japan). Unstained cells were considered viable, whereas blue-stained cells were considered non-viable. The total number of viable cells per well was calculated and plotted.

#### 4.3.3. Dose–Response Curves

Dose–response curves were obtained by nonlinear regression using a four-parameter logistic equation (GraphPad Prism 8):

*Y = Bottom + (Top − Bottom)/(1 + 10^((LogIC50 − X) × HillSlope)).*

where *X* is the logarithm of the drug concentration.

### 4.4. MitoTracker™ Red CMXRos Staining

Mitochondrial signal and cell density were assessed using MitoTracker™ Red CMXRos and DAPI fluorescence. MDA-MB-231 cells were seeded at 2 × 10^3^ cells/well in 96-well plates and incubated for 18 h to allow stable attachment. After drug treatment for 72 h, the culture medium was removed and cells were gently washed once with Hank’s balanced salt solution (HBSS). MitoTracker™ Dyes for Mitochondria Labeling (Thermo Fisher Scientific, St. Louis, MO, USA) was diluted to a final concentration of 200 nM in HBSS and added to each well, followed by incubation for 30 min at 37 °C in the dark. Cells were then washed once with HBSS, and nuclei were counterstained using an antifade mounting medium containing DAPI (VECTASHIELD^®^ Vibrance, Burlingame, CA, USA) according to the manufacturer’s instructions. Fluorescence was measured in the same plate using a Fluoroskan FL microplate fluorometer (Thermo Scientific, St. Louis, MO, USA) controlled by SkanIt Software 6.1.1 (Thermo Scientific, St. Louis, MO, USA). DAPI fluorescence was recorded at an excitation/emission of 364/460 nm, and MitoTracker™ Dyes fluorescence at 584/612 nm. Background-subtracted fluorescence values were normalized to the mean of the corresponding vehicle-treated control group and expressed as percentages of control for each readout.

### 4.5. Calcium Imaging

Calcium responses in the MDA-MB-231 cell line were measured using the fluorescent calcium indicator Fura-2 AM (Invitrogen, Carlsbad, CA, USA) dye. Cells were loaded with 5 µM Fura-2 AM in HBSS containing 0.1% Pluronic F-127 for 30 min at 37 °C. After washing, fluorescence was recorded using an inverted fluorescence microscope (BX-70, Olympus, Tokyo, Japan) following stimulation with 100 µM histamine. Fluorescence intensity changes were analyzed using MetaFluor^®^ Software (Version 8.0, Molecular Devices, Villepinte, France).

### 4.6. RNA Interference

Small interfering RNAs (siRNAs) targeting human HRH1 (siHRH1) and human ARF1 (siARF1) were purchased as AccuTarget™ Predesigned siRNA from Bioneer (Seoul, Korea). A non-targeting control (siCTRL) was purchased from Dharmacon (Lafayette, CO, USA). The sequences for siHRH1 and siARF1 were 5′-GUAGUUUGGAAAGUUCUUA-3′ and 5′-CUGCAUUCCAUAGCCAUGU-3′, respectively. Cells were transfected with 50 nM siRNA using Lipofectamine 2000 (Invitrogen, Carlsbad, CA, USA) according to the manufacturer’s instructions for siRNA-mediated gene silencing. At 48 h post-transfection, cells were treated with azelastine (30 µM or 50 µM), and viability was assessed 72 h later.

### 4.7. Bioinformatic Analysis of Patient Datasets

#### 4.7.1. Gene Expression Analysis

Differential expression of HRH1 and ARF1 across normal, tumor, and metastatic breast tissues was analyzed using TNMplot (https://tnmplot.com/analysis/ (accessed on 15 September 2025). This platform utilizes RNA-seq data from The Cancer Genome Atlas (TCGA) and the Genotype-Tissue Expression (GTEx) projects. The TCGA breast cancer (BRCA) dataset within the TIMER 2.0 platform (http://timer.cistrome.org/ (accessed on 15 September 2025) was also used to analyze expression levels across different molecular subtypes.

#### 4.7.2. Patient Cohort and Survival Analysis

Survival analysis in TNBC patients was performed using the Cancer Target Gene Screening (CTGS) program (http://ctgs.biohackers.net/ (accessed on 15 September 2025), which is based on the Molecular Taxonomy of Breast Cancer International Consortium (METABRIC) cohort. From this cohort, a total of 299 TNBC patients were included for Kaplan–Meier analysis. The clinical characteristics of these patients were as follows: the median age was 55 years (range, 28–89 years), with a distribution by tumor stage of Stage I (17%), Stage II (55%), and Stage III (23%). For the analysis, patients were stratified into high- and low-expression groups based on the median expression value of the gene of interest. Overall Survival (OS) and Disease-Free Survival (DFS) curves were generated, and differences were assessed using the log-rank test.

### 4.8. Quantitative Real-Time PCR

Total RNA was isolated from MDA-MB-231 cells following siRNA transfection using RNAiso Plus (TAKARA BIO, Shiga, Japan) according to the manufacturer’s instructions.

For cDNA synthesis, Oligo dT (Qiagen, Germantown, MD, USA) primers were annealed to the isolated total RNA. The reverse transcription reaction was then performed using ImProm-II™ Reverse Transcriptase (Promega, Madison, WI, USA) following the conditions specified by the supplier.

The synthesized cDNA samples were subjected to qRT-PCR using 2x SYBR GREEN NO-ROX KIT (BIOLINE, Gyeonggi-do, Korea) on a real-time PCR system. The gene expression levels were quantified using the following primer sequences:

Human β-actin
F-CACCATGGATGATGATATCGC, R-CACATAGGAATCCTTCTGACCCA
Human HRH1
F-GGATGCCAAGAAACCAGGGAAG, R-CTCTTGGCTGAAGACAACTGGGG
Human ARF1
F-CTCACTACGCCACAGGAACT, R-CAGCCAGTCCAGTCCTTCAT

β-actin was used as an endogenous reference gene to normalize the expression levels of HRH1 and ARF1.

### 4.9. Western Blot

MDA-MB-231 cells were treated with azelastine for 72 h and subsequently lysed in lysis buffer to prepare whole-cell lysates. The lysates were centrifuged at 14,000× *g* for 15 min at 4 °C, and the supernatants were collected. Protein concentrations were determined using the Bradford assay. Equal amounts of protein (5 µg per sample) were separated by SDS-PAGE on 12% polyacrylamide gels and transferred onto PVDF membranes using a wet transfer system (250 mA, 30 min).

Membranes were blocked with 5% non-fat skim milk in TBST for 1 h at room temperature and incubated overnight at 4 °C with the following primary antibodies: p-ERK (1:2000; Cell Signaling Technology, Danvers, MA, USA), ERK (1:2000; Cell Signaling Technology), and ACTIN (1:5000; Santa Cruz Biotechnology, Dallas, TX, USA). After three washes in TBST, membranes were incubated with HRP-conjugated secondary antibodies (anti-mouse IgG, 1:2000 for p-ERK; anti-rabbit IgG, 1:2000 for ERK; anti-mouse IgG, 1:10,000 for ACTIN) for 1 h at room temperature.

Protein bands were detected using an enhanced chemiluminescence (ECL) detection system (Thermo Fisher Scientific) and visualized with the ChemiDoc XRS+ imaging system (Bio-Rad). Densitometric quantification was performed using ImageJ software 1.54 (NIH), and band intensities were normalized to ACTIN to ensure equal protein loading.

### 4.10. Statistical Analysis

Data are presented as mean ± SD from ≥3 independent biological replicates (independent wells/plates from separate experiments; the well/plate is the unit of analysis). Tests were selected based on design: unpaired two-tailed Student’s *t*-test for two-group comparisons (Figure 3C,D and Figure 5A,B,F); one-way ANOVA with Dunnett’s multiple comparisons test for ≥3 levels of a single factor at a single time point with control-focused contrasts (Figure 1D,E); and two-way ANOVA with Tukey’s multiple comparisons test when two factors and their interaction were of interest (Figure 3B,E, Figure 5C–E and Figure 6A,B). Survival curves were analyzed using the Kaplan–Meier method and log-rank test. *p*-values less than 0.05 were considered statistically significant. Analyses were conducted using GraphPad Prism 8.0 (GraphPad Software, San Diego, CA, USA).

### 4.11. Ethics Statement

This study utilized publicly available, de-identified patient data obtained from TCGA, TIMER2.0, and TNMplot databases. As all data were previously collected and anonymized, ethical approval and informed consent were not required under institutional and international guidelines. All experimental procedures using cell lines complied with institutional biosafety and research policies.

## 5. Conclusions

Azelastine suppresses TNBC cell viability through an HRH1-independent, ARF1-dependent mechanism with downstream attenuation of the ERK–Drp1 axis, a finding demonstrated in MDA-MB-231 cells. These data, together with prior evidence that azelastine can directly engage ARF1, nominate ARF1 as a tractable vulnerability in TNBC and support the repurposing of azelastine as a mechanism-based therapeutic candidate. In the current treatment landscape—dominated by PD-1 blockade, PARP inhibition for gBRCA-mutated disease, and TROP-2-directed ADCs—targeting the ARF1 → ERK → Drp1 signaling hub offers a complementary, biomarker-refinable strategy. Prospective studies should validate efficacy in vivo, define pharmacodynamic markers along the ARF1–ERK–Drp1 pathway, and evaluate rational combinations and patient selection based on ARF1 status.

## Data Availability

The data presented in this study are available in publicly accessible repositories. The datasets analyzed during the current study were obtained from TCGA (https://www.cancer.gov/tcga (accessed on 15 September 2025) and METABRIC (https://ega-archive.org/ (accessed on 15 September 2025).

## Abbreviations

The following abbreviations are used in this manuscript:

ARF1	ADP-ribosylation Factor 1
GBF1	Golgi-specific brefeldin A-resistance guanine nucleotide exchange factor 1
GCA	Golgicide A
ERK	Extracellular signal-regulated kinase
PI3K	Phosphoinositide-3-kinase
AKT	Protein Kinase B
HRH1	Histamine Receptor 1
Drp1	Dynamic-related protein 1
DAPI	4′,6-diamidino-2-phenylindole
TNBC	Triple-Negative Breast Cancer
CTGS	Cancer Target Gene Screening
TCGA	The Cancer Genome Atlas
